# Human augmentation, not replacement: A research agenda for AI and robotics in the industry

**DOI:** 10.3389/frobt.2022.997386

**Published:** 2022-10-04

**Authors:** Sarah Dégallier-Rochat, Mascha Kurpicz-Briki, Nada Endrissat, Olena Yatsenko

**Affiliations:** ^1^ Institute for Human-Centered Engineering, School of Engineering and Computer Science, Bern University of Applied Sciences, Bern, Switzerland; ^2^ Institute for Data Application and Security, School of Engineering and Computer Science, Bern University of Applied Sciences, Bern, Switzerland; ^3^ Institute New Work, Business School, Bern University of Applied Sciences, Bern, Switzerland

**Keywords:** human-machine interaction, artificial intelligence, augmented intelligence, robotics, complementary cooperation

## Introduction

When talking about the threats of work automation through robotics and/or AI, the topic of human replacement is often the first to show up. If it is sometimes seen as something positive, it often revives the collective fear of people losing their jobs, a fear that has been continuously entertained through political discourse against immigration (Goldberg, 2015). The difference being that the threat is now machine that is thought to be much more productive than humans or, even, on the verge of becoming more intelligent than them: The so-called technology singularity (Kurzweil, 2005). In this position paper, we argue that the singularity myth has a negative influence on the current research agenda in artificial intelligence (AI) and robotics. Indeed, if complete human replacement is more a myth than a reality, new technologies are altering the way that we work, posing new challenges for the way we manage human-machine interactions, including work alienation, decision-making power and fairness that require attention. We call for greater attention to augmentation technologies that empower humans rather than mechanize and deskill them. We lay out the advantages of such a path, stressing that the industry can truly benefit from new technologies when human-machine complementarity is leveraged.

## Human replacement is not the main threat

To better understand the general skepticism towards the singularity, it is useful to make a distinction between “narrow artificial intelligence” (NAI) which aims at efficiently solving a complex problem and “artificial general intelligence” (AGI) which aims at reproducing human intelligence capabilities (Goertzel, 2007). Both forms of artificial intelligence are based on very different grounds, with most of the research efforts focusing on NAI (Bundy, 2017). AGI, on the contrary, is “*still at the stage of infancy*” and most of the contributions in the field rely more on “*imagination than on trustworthy data*” (Braga and Logan, 2019). Indeed, there is no proof that the singularity really exists, and if it does, it is very unlikely to happen in a near future (National Science and Technology Council, 2016).

However, despite the highly hypothetical nature of the singularity, there is a lot of discussion around it, which has led to the creation of a modern myth, sometimes referred to as Apocalyptic AI (Geraci, 2008). Indeed, the hope to create a perfect, immortal human being is strongly anchored in the idea of human self-deification (Zimmermann, 2008). Interestingly, the tenets of this myth have been mainly sustained by experts in the fields (Natale and Ballatore, 2020) and it is still strongly influencing current scientific research and perception of AI (Bringsjord et al., 2012). One of most pregnant myths is the one of full autonomy, as described by (Mindell, 2015): contrarily to this myth, robots and (N) AI will never be completely autonomous, because, *per design*, intentions will always need to be defined by humans, ruling out the possibility of complete human replacement.

Nevertheless, the myth of full autonomy is strongly present in *both the public and scientific debate*. For instance, in 2017, a Eurobarometer survey showed that 72% of Europeans believe that “*robots and AI steal people’s job*” (European Commission, 2017). In 2017, a study from Frey and Osbourne (Frey and Osborne, 2017) predicted that 47% per cent of the jobs in the USA were at high risk to disappear through automation. Another study (Arntz et al., 2016) concluded that 9% of the jobs could be automatable, but not necessarily in an economically viable way. The main difference between the two studies is that Frey and Osbourne explored which tasks could in principle be automated, while overlooking the fact that these tasks were part of a more complex job that in its entirety was not suitable for automation (Fernandez-Macias and Bisello, 2020; Parker and Grote, 2022). As pointed out in (Macias et al., 2016; Autor and Salomons, 2018), previous waves of industrialization have already led to the automation of most of the physical labor and the remaining tasks are completely beyond the current capabilities of robots and (N)AI. In 2020, in a report of the European commission (Klenert et al., 2020), a systematic study of the impact of automation on jobs between 1995 and 2015 in Europe was performed, leading to the conclusion that automation had a positive impact on employment in manufacturing. The conclusions found in the literature on job replacement are however mitigated, due to differences in methodologies and level of analysis. A generic survey (Barbieri et al., 2019) highlighted that more detailed analyses considering the difficulties of automation lead to far less pessimistic predictions, comparable to previous technological revolutions (Dahlin, 2019). While the risk of unemployment should not be underestimated, we believe that the main challenge with AI and robotics lies in the quality of the interaction with the machine.

## Human-machine interaction: Mechanization or empowerment?

If new automation technologies are unlikely to replace us in the near future, they are going to alter the way we work (Brynjolfsson and McAfee, 2011). Indeed, with the increasing data processing power of technology, machines can exercise intentionality over protocols and action selection, thereby challenging the dominance of human agency, autonomy and, ultimately, power (Murray et al., 2021). This calls for greater attention to the potential of worker empowerment or mechanization, shifting the focus from artificial intelligence to augmented intelligence.

In order to address this issue, we build on the taxonomy of conjoined agency defined as “*shared capacity between humans and nonhumans to exercise intentionality*” (p. 555) by Murray and others (Murray et al., 2021). Agency over *what to do* (action selection) and *how to do* it (protocol development) can rest either with the human or with the technology leading to four forms of conjoined agency including assisting, augmenting, arresting, or automating technologies (Table 1). We believe that, if the agency over action selection rests with the human, the interaction with the machine can help empowering them. However, if the agency for action selection gets transferred to technology, we witness a form of human mechanization through the interaction (Bui, 2020).

**TABLE 1:** Empowerment and mechanization in human-machine interactions (adapted from Murray et al., 2021, p. 555).

		Locus of agency in protocol development	
		Human	Technology
**Locus of Agency in Action Selection**	**Human**	Assisting technologies	Augmenting technologies
		Empowerment	Empowerment
	**Technology**	Arresting technologies	Automating technologies
		Mechanization	Mechanization

In the following, we highlight the role of the nature of the interaction for our understanding of augmented intelligence and augmented worker.

### Augmented intelligence

Augmented Intelligence refers to technologies of Artificial Intelligence that do not replace human decision-making, but rather provide additional information for decision-support (*What is Augmented Intelligence?—* IEEE, 2022), and thus falls in the above category of *Empowerment*. Imagine an AI-based software automatically extracting the relevant information from thousands of pages of text, and a human deciding upon that, including the broader view with more context, and reflecting the decision. The data analysis task would take days for a human, and the machine would not be able to consider any other aspects than the narrow view on the data. Augmented Intelligence thus allows for faster and efficient human decision-making. However, there are still challenges when implementing such technology; the decision-making may result in unfair and discriminatory decisions, but also in workers not being able to contest the decision because of its lack of explainability, leading to a loss of employees’ autonomy and job control (Jankauskaitė et al., 2022). Similarly, AI-based worker management (AIWM) systems (European Agency for Safety and Health at Work, 2022) can be seen as *empowering* technology for the manager, but the worker might be subject to a form of *mechanization* highlighting the need to reflect on power-asymmetries that new technology can perpetuate. Likewise, algorithmic and augmenting systems can accentuate discrimination, ranging from unfair decisions for specific groups in risk assessment systems or exclusion from the labor market (O’Neil, 2016; Draeger and Müller-Eiselt, 2019; Eubanks, 2019), to systems not working (or not working properly) for specific groups. This calls for a sensitive and reflective practice of using data and augmented intelligence in decision-making processes including a reflection on the moral values with which we, as a society, want to shape the future of human-machine interactions. When decisions are taken by machines, specific assessments can be implemented that require human intervention to assess fairness and human integrity (Leicht-Deobald et al., 2019). Indeed, it has been argued that it is a crucial yet neglected aspect of fairness metrics to consider the moral perspective (Leicht-Deobald et al., 2019; Hertweck et al., 2021), which cannot be automated. The decision what forms of discrimination, marginalization, and exclusions are morally justified, ultimately rests with the human.

### Augmented worker

**FIGURE 1:**
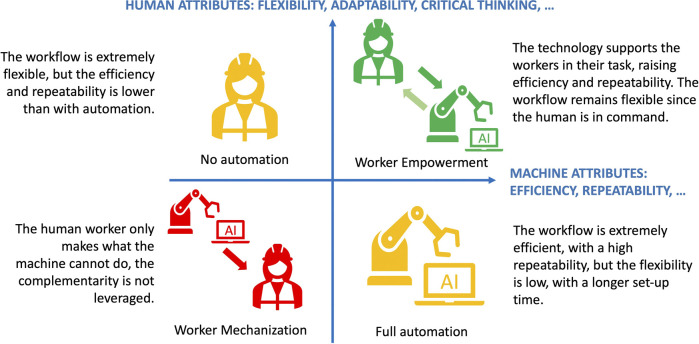
The four types of workflows: Manual work, full automation, worker empowerment and worker mechanization. The two axes show the strengths of both humans and machines and the characteristics of the workflow associated with different forms of human-machine interaction.

The notion of Augmented Worker or Operator 4.0 (Romero et al., 2020) refers to an anthropocentric approach to manufacturing (Dworschak and Zaiser, 2014): The machine is seen as a tool to *empower* the workers, rather than replace them. Indeed, the world of industrialization is experiencing a change of paradigm: while the focus has been on human replacement for decades, it is now becoming clear that humans will still be needed in factories in the foreseeable future (Tan et al., 2019; Romero et al., 2020). To cope with the growing versatility of the market, some authors argue further that companies can achieve the largest boosts in performance by leveraging human-machine complementary strengths (Daugherty and Wilson, 2018): The flexibility of human work combined with the efficiency of automation. However, in practice, most manufacturing system are still implemented based on a traditional, technocentric approach (Dworschak and Zaiser, 2014): The work process is determined by the technology. The focus is on performance and repeatability and the human is expected to work as a machine (Bui, 2020), possibly leading to a feeling of *mechanization*. Several studies have underlined the danger of a further polarization of the work market (Holm et al., 2020): The people that serve the technology, the cyberproletariat (Huws, 2014) and the ones that detain the skills to control the technology. However, if in the past, machines were complex and costly, and the workers had little possibilities to modify the machines or to complement their own skills (Levine, 2019), new technologies such as collaborative robotics are democratizing the technology and making it more accessible to non-experts (Villani et al., 2018). AI and robotics become tools to support the *augmented workers* to make them more efficient in their jobs. Rather than reducing the decision control of the workers to avoid errors, the technology is used to prevent their errors and assist them in their task (Lu et al., 2021). According to manufacturing approaches such as Kaizen (Janjić et al., 2020) and Agile (Gunasekaran et al., 2019), it is only by giving control back to the worker on the shop floor that the full potential of new technologies can be leveraged to boost productivity, motivation and innovation, as illustrated in Figure 1.

## Conclusion

The idea of a singularity outperforming humans continues to fascinate researchers and practitioners alike. In this position paper, we argued that the question of singularity is misleading. We need to rather attend to questions that are at the heart of our daily interactions with machines, shifting from the question of human replacement to the question of the quality of the human-machine interaction. Taking a closer look at the nature of such conjoined agency, we have differentiated among interactions that tend to mechanize and those that empower the human. To create viable futures, the emphasis should be on the latter. We have highlighted an agenda to bring this potential to fruit including questions of fairness, discrimination, skill, and power in organizations. Ultimately, it is not the technology itself that creates a threat for humans, but rather the way it is implemented (Clegg, 2000; Moore, 2019). We therefore call more attention to questions of agency and power when designing technology to ensure a vital *human-in-command* approach (De Stefano, 2018) to create sustainable employment and learning opportunities for all.
